# Diaphragm electrical activity monitoring as a breakpoint in the management of a tetraplegic child

**DOI:** 10.1186/s13054-017-1702-5

**Published:** 2017-05-26

**Authors:** Guillaume Mortamet, François Proulx, Benjamin Crulli, Nadia Savy, Philippe Jouvet, Guillaume Emeriaud

**Affiliations:** Paediatric Intensive Care Unit, CHU Sainte-Justine, 3175 Côte Sainte-Catherine, Montreal, QC H3T 1C5 Canada

**Keywords:** Mechanical ventilation, Electrical activity of the diaphragm, Diaphragm function, Pediatric intensive care unit, Pediatrics

Over the last decade, new technology has been developed to continuously record the electrical activity of the diaphragm (EAdi) at the bedside [1]. EAdi monitoring has been shown to be useful in assessing the patient’s ventilatory drive, in adjusting ventilatory support, and in detecting patient–ventilator asynchrony [2–4]. In the present case, we highlight how monitoring EAdi could be a sensitive diagnostic tool to detect spontaneous respiratory cycles in a mechanically ventilated child with tetraplegia.

An 8-year-old girl was admitted to our pediatric intensive care unit (PICU) for a rapidly progressive right hemiparesis. The CT scan revealed a large C3–C4 medullary arteriovenous malformation predominantly. An urgent embolization was attempted, but severe edema and hemorrhagic transformation of venous thrombosis developed, leading to tetraplegia with dysautonomia. She underwent tracheostomy on day 12 due to the absence of spontaneous breathing. Three months later, an MRI scan showed extensive cervical cord fibrosis and atrophy at C2–C3–C4 levels (Fig. 1). On day 90, a phrenic nerve stimulation test was conducted to assess the potential for diaphragmatic pacing. No esophageal pressure deflection was induced by the stimulation. However, after a few respiratory pauses applied for the stimulation, we noted some spontaneous cycles on the EAdi recordings (about 5 μV) associated with esophageal pressure deflections (5–10 cmH_2_O). Continuous monitoring of EAdi was performed while decreasing the level of ventilator support, thereby confirming an intermittent and small respiratory drive (Fig. 2). Weaning using NAVA was started in order to favor the patient’s own respiratory drive, which gradually increased over time (Fig. 2). She was progressively and successfully weaned from the ventilator during daytime on day 162 and the patient was discharged home on day 374.

**Fig. 1:**
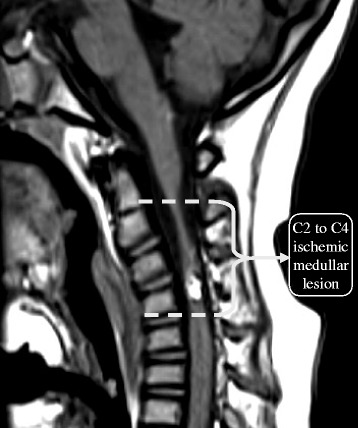
Brain MRI image (T2-weighted) performed on day 90 showing extensive cervical cord fibrosis and atrophy, which was more severe at C2–C3–C4 levels

**Fig. 2:**
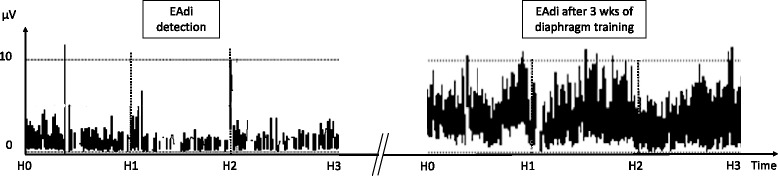
Evolution of diaphragm electrical activity (*EAdi*) over 3-hour recordings﻿ (H0 to H3) at the time of diagnosis of the present ventilatory drive (*left panel*), showing intermittent EAdi at low levels, to 3 weeks (*wks*) later (*right panel*), after weaning in NAVA, showing sustained and higher levels of EAdi

Although it was not observed clinically, residual respiratory activity was evidenced by the EAdi monitoring. We hypothesize that complete ventilatory support during the first 3 months may have induced some diaphragmatic dysfunction [5], making it difficult to detect a respiratory drive. While we were initially considering the implantation of a diaphragmatic pacing device, we instead opted for a ventilation weaning challenge using NAVA, which allowed a gradual decrease in the level of support while preserving spontaneous breathing and diaphragm training.

This case illustrates that clinical assessment could lack sensitivity in detecting spontaneous breathing in patients with low respiratory drive. EAdi monitoring may be considered to precisely assess the presence of spontaneous breathing in complex patients, especially before making important management decisions.

## Abbreviations

CT: Computerized tomography
EAdi: Electrical activity of the diaphragm
MRI: Magnetic resonance imaging
NAVA: Neurally adjusted ventilatory assist
PICU: Pediatric intensive care unit
